# Miles down for lunch: deep-sea *in situ* observations of Arctic finned octopods *Cirroteuthis muelleri* suggest pelagic–benthic feeding migration

**DOI:** 10.1098/rspb.2023.0640

**Published:** 2023-06-28

**Authors:** Alexey V. Golikov, Julian B. Stauffer, Sophie V. Schindler, James Taylor, Lilian Boehringer, Autun Purser, Rushan M. Sabirov, Henk-Jan Hoving

**Affiliations:** ^1^ GEOMAR Helmholtz Centre for Ocean Research Kiel, 24105 Kiel, Germany; ^2^ Senckenberg am Meer, German Centre for Marine Biodiversity Research, c/o Biocenter Grindel, Center of Natural History, Universität Hamburg, 20146 Hamburg, Germany; ^3^ Alfred Wegener Institute, Helmholtz Centre for Polar and Marine Research, 27570 Bremerhaven, Germany; ^4^ Department of Zoology, Kazan Federal University, 420008 Kazan, Russia

**Keywords:** cephalopoda, predator, behaviour, locomotion, remotely operated vehicle, PELAGIOS

## Abstract

Deep-sea cephalopods are diverse, abundant, and poorly understood. The Cirrata are gelatinous finned octopods and among the deepest-living cephalopods ever recorded. Their natural feeding behaviour remains undocumented. During deep-sea surveys in the Arctic, we observed *Cirroteuthis muelleri*. Octopods were encountered with their web spread wide, motionless and drifting in the water column 500–2600 m from the seafloor. Individuals of *C. muelleri* were also repeatedly observed on the seafloor where they exhibited a repeated, behavioural sequence interpreted as feeding. The sequence (11–21 s) consisted of arm web spreading, enveloping and retreating. Prey capture happened during the enveloping phase and lasted 5–49 s. Numerous traces of feeding activity were also observed on the seafloor. The utilization of the water column for drifting and the deep seafloor for feeding is a novel migration behaviour for cephalopods, but known from gelatinous fishes and holothurians. By benthic feeding, the octopods benefit from the enhanced nutrient availability on the seafloor. Drifting in the water column may be an energetically efficient way of transportation while simultaneously avoiding seafloor-associated predators. *In situ* observations are indispensable to discover the behaviour of abundant megafauna, and the energetic coupling between the pelagic and benthic deep sea.

## 1. Introduction

The bathypelagic zone (1000–4000 m) is the largest and least explored biome on Earth [1,2]. It comprises over 75% of the total ocean volume [1]. Most knowledge on bathypelagic fauna is available from net sampling [1,2]. However, this method is not well suited for sampling of large-sized, delicate and fragile invertebrates and fishes which may become damaged beyond identification and quantification [3–6]. The observations of organisms in their natural habitat (*in situ* observations) via camera systems or submersibles have brought significant insights in deep-sea diversity and ecosystem functioning, e.g. [7,8]. However, due to financial and logistical challenges of deep-sea research, and the difficulty of observing animals in their natural habitat without anthropogenic influence, basic biological information, including feeding behaviour, remains sparse for many abundant taxa.

Cephalopod molluscs are abundant and diverse in the deep sea [9]. They are important prey for many oceanic consumers including top predators [10–14]. Cephalopods prey on crustaceans, fishes and other cephalopods [13,15]. Many oceanic squids optimize their foraging by daily vertical migration [13,16]. They occur at mesopelagic depths during the day and migrate to the epipelagic zone at night to benefit from enhanced surface productivity, while simultaneously avoiding predation by visually attuned predators [13,16]. Deep-sea surveys with remotely operated vehicles (ROVs) have resulted in discoveries of interesting and unusual feeding behaviours in deep-sea cephalopods [17–19]. However, the feeding behaviour of many deep-sea cephalopod species has never been observed [9].

Cirrata are finned octopods that occur in the deep seas of all oceans [3,20]. As prominent deep-sea fauna, they have been filmed and photographed since the 1970s (review, [3]), and are the deepest-dwelling cephalopods (6957 m, Indian Ocean) [21]. Their most distinct external characteristics are a semi-gelatinous body consistency, a well-developed web and paired, finger-like projections called cirri that are situated in between suckers [3,20]. Cirrate octopods are easily recognized by a pair of fins on the body, resembling the ears of the flying elephant in *Dumbo*, the Disney movie of 1941, which has resulted in the name ‘dumbo’ or ‘jumbo' octopus being commonly used for some cirrate taxa [3]. The limited stomach contents data of cirrate octopods report a diet of predominantly Crustacea and Polychaeta, and occasional records of Gastropoda, Bivalvia, Cephalopoda and Osteichthyes [3,22–25]. The only observations of confirmed feeding behaviour in cirrates are based on a single female of *Opisthoteuthis* sp. that was kept in an aquarium for 53 days [3,26].

During deep-sea surveys with ROV and towed cameras in the Arctic, we repeatedly observed individuals of *Cirroteuthis muelleri* Eschricht, 1836 [27] (family Cirroteuthidae), the only species of cirrate octopods described from the Arctic Ocean [15,27–29]. Octopods were drifting in the water column at 500–2600 m from the seafloor. We also observed *C. muelleri* on the seafloor, and using close up ROV observations we describe the first observations of cirrate feeding behaviour under natural conditions. The specific behaviours associated with the pelagic and benthic habitat strongly suggest that *C. muelleri* migrates from the water column to the seafloor to feed. Pelagic–benthic feeding migration is a novel behaviour for cephalopods, but is known in deep-sea gelatinous fishes and holothurians [30,31]. We discuss our observations in the context of convergent evolution in deep-sea taxa, and the ecological linking of benthic and pelagic habitats.

## 2. Material and methods

**Figure 1.**
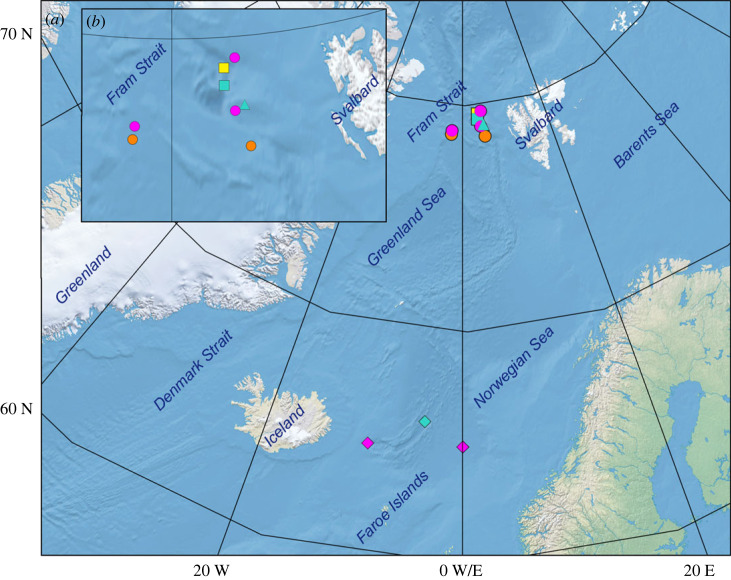
(*a*) A map with the geographical locations of our *in situ* video observations of *Cirroteuthis muelleri* in the Fram Strait and Norwegian Sea, and (*b*) the Fram Strait with these locations in higher resolution. Square, ROV *Aurora Borealis*; triangle, ROV *Phoca*; round, PELAGIOS; diamond, ROV *Kiel 6000*; magenta, drifting; orange, drifting + fin-swimming; yellow, fin-swimming; turquoise, feeding + fin-swimming.

Observations in the Fram Strait were collected during the cruises on R/Vs *Polarstern* (PS121, 2019, and PS126, 2021), *Maria S. Merian* (MSM95, 2020 and MSM108, 2022) and *Kronprins Haakon* (HACON21, 2021) [32]. During PS121 and PS126, we used the modified Pelagic In situ Observation System (PELAGIOS) [5]. This instrument consists of a forward-viewing deep-sea camera with LED illumination, and a depth sensor (CTD or Star-Oddi). All components are mounted on a steel frame. The PELAGIOS was towed during horizontal transects (ship speed 0.5 m s^−1^) at depths down to 2400 m for 10–20 min per transect. During PS121, ROV *Phoca* [33] recorded one cirrate on the seafloor (figure 1; electronic supplementary material, table S1). During the HACON21 cruise, ROV *Aurora Borealis* recorded two cirrate observations [32] (figure 1; electronic supplementary material, table S1). During cruises on R/Vs *Polarstern* (PS121 and PS126) and *Maria S. Merian* (MSM95 and MSM108) in the Fram Strait, and the *Kronprins Haakon* (HACON19, 2019) cruise across the Aurora mound on the Gakkel Karasik Ridge (figure 1; electronic supplementary material, table S1), the Ocean Floor Observation and Bathymetry System (OFOBS) towed camera sled [34] was used to visually survey the seafloor. Observations in the Norwegian Sea were collected during a cruise on R/V *Sonne* (SO276, 2020) using ROV *Kiel 6000* [35] (figure 1; electronic supplementary material, table S1). Specifically targeted observations by ROVs are referred to as ‘long observations', and PELAGIOS and ROV observations during the deployment/recovery are referred as ‘short observations' (electronic supplementary material, table S1).

This study uses cut-out video sequences and frames of cirrates from the videos, as well as still images collected using the OFOBS towed camera system. Details of full video annotations and analyses from R/Vs *Polarstern* and *Sonne* can be found in Stauffer [36]. During observations by ‘ROV Aurora Borealis', two lasers with a distance of 16 cm were used as underwater scale, and allowed size reconstruction. In all OFOBS images, three red laser points are visible in the central area of each image. These laser points have a 50 cm spacing, and allow for the computation of image area using the PAPARA(ZZ)I software [37]. Depth data from PELAGIOS observations were either taken from cable length noted in the station protocol or are recalculated from pressure logger data via https://bluerobotics.com/learn/pressure-depth-calculator/ (electronic supplementary material, table S1). Bottom depths of the respective locations are found via https://www.ncei.noaa.gov/maps/bathymetry/ using the coordinates for all PELAGIOS stations except PS121-EG4-video1, where bottom depth is recorded onboard (electronic supplementary material, table S1).

We reconstructed absolute and relative proportions of cirrate body parts (and absolute sizes for the observations by ROV *Aurora Borealis*) and compared those to Golikov *et al.* [22]. Morphometric measurements included: mantle length (ML), total length (TL), ML relative to TL (%), fin length and width, eye and sucker diameter, and cirri length relative to ML (%) (electronic supplementary material, table S2). A Mann–Whitney *U*-test and a Kruskal–Wallis *H*-test with a post-hoc Dunn's *Z-*test [38] in PAST 4.02 [39] were used for comparison among these characters (electronic supplementary material, table s3). The value of *α* ≤ 0.05 is considered significant in this study. Behaviour interpretation followed the review of Collins & Villanueva [3].

## 3. Results

### (a) Morphology, identification and habitat parameters

Absolute sizes and relative proportions of the observed octopods fit the *C. muelleri* populations of Iceland, West Greenland or the Barents Sea slope (electronic supplementary material, table S2). The only exception is relative fin width, which is wider than previously known in seven of eleven individuals where measurements are possible (electronic supplementary material, table S2). Our individuals are smaller (both ML and TL) than other North Atlantic cirrates, *Cirrothauma murrayi* Chun, 1911 [40] and *Ci. magna* (Hoyle, 1885 [41]) [42–44] (electronic supplementary material, table S3). After fixation, cirrate octopods shrink and their morphology distorts [3,6]. The smaller sizes of our individuals maintained despite fixation shrinkage of the both other species used for comparison. Other parameters, such as fins and eyes morphometry (electronic supplementary material, table S3), may potentially be biased due to the shrinkage and should be considered with care. Observation depths are 522–4270 m, and bottom depths at these locations are 1506–4270 m (electronic supplementary material, table S1). Bottom temperatures are −0.72 to 0.10°C (electronic supplementary material, table S1).

### (b) Behaviour

#### (i) Feeding

**Figure 2.**
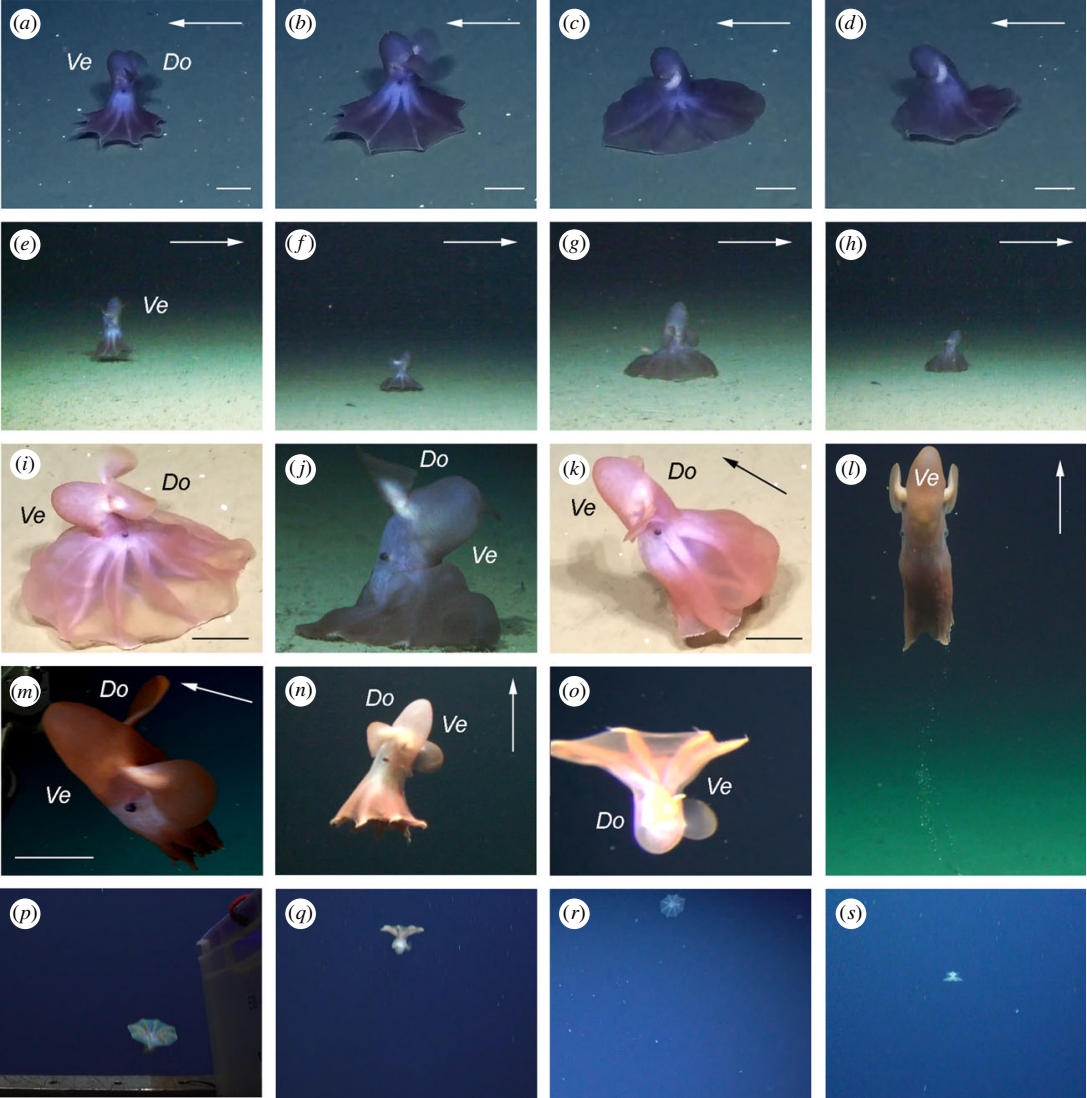
*In situ* observations of *Cirroteuthis muelleri*. (*a,e*) Initial pose/pause in the feeding behavioural sequence, HACON21-ROV019 (*a*) and PS121-ROV001 (*e*). (*b,f*) Spreading phase in the feeding behavioural sequence, HACON21-ROV019 (*b*) and PS121-ROV001 (*f*). (*c,g*) Enveloping phase in the feeding behavioural sequence, HACON21-ROV019 (*c*) and PS121-ROV001 (*g*). (*d,h*) Retreating phase in the feeding behavioural sequence, HACON21-ROV019 (*d*) and PS121-ROV001 (*h*). (*i,j*) Prey capture, HACON21-ROV019 (*i*) and PS121-ROV001 (*j*). (*k*) Take-off from the seafloor, HACON21-ROV019. (*l,m*) fin-swimming, 037ROV02-tROVobs-LowerHD (*l*), note sand and slime falling from the octopod, and HACON21-ROV003 (*m*). (*n*) Possible aborted protective reaction or pelagic take-off attempt, PS121-ROV001. (*o–s*) Drifting, PS121-HG4-video2 (*o*), 004ROV01-video1 (*p,q*), PS126-EG4-video2 (*r*), 054ROV03-video1 (*s*). Arrow shows movement direction, where applicable. Ve, ventral; Do, dorsal. Scale bars = 100 mm.

**Figure 3.**
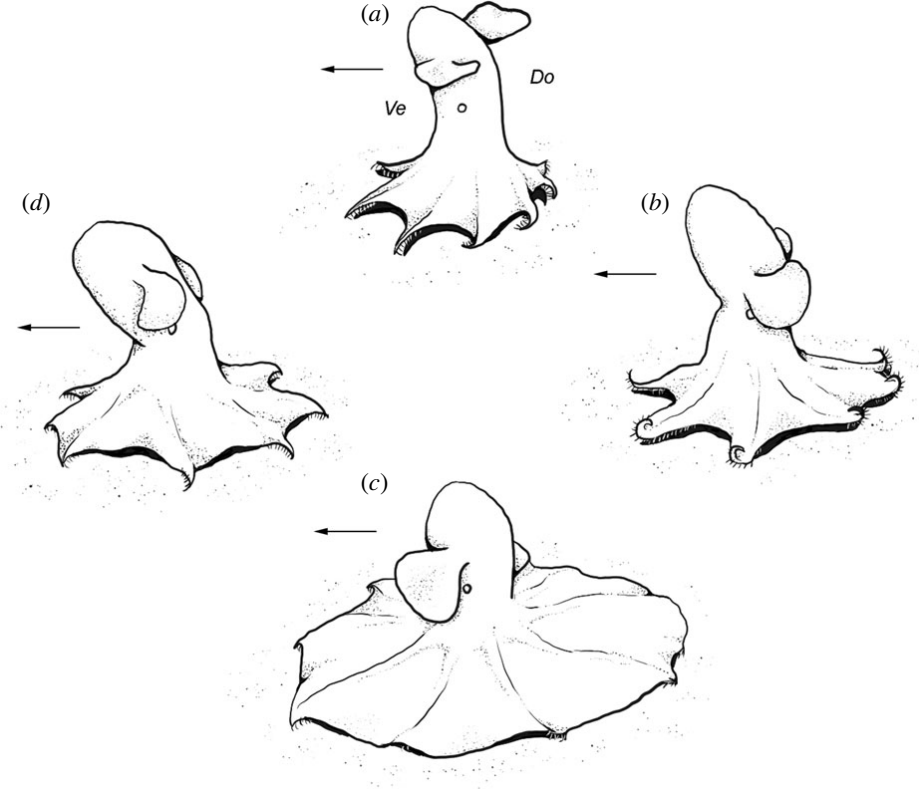
Schema of the feeding behavioural sequence in *Cirroteuthis muelleri*. (*a*) Initial pose/pause, (*b*) spreading phase, (*c*) enveloping phase, (*d*) retreating phase. Arrow shows movement direction. Ve, ventral; Do, dorsal.

We observed three cirrates exhibiting presumed feeding behaviour on the seafloor of the Fram Strait (1506 and 3693 m) and the Norwegian Sea (3615 m) (figure 1; electronic supplementary material, table S1). The feeding behaviour consists of a repetition of the same sequence of movements (figures 2*a–h* and 3; table 1; electronic supplementary material, videos S1–S3). The octopod fin-swims slowly just above the seafloor with the speed of 4–8 fin stroke cycles min^−1^, while performing this behaviour (figures 2*a–h* and 3; electronic supplementary material, videos S1–S3). The octopod keeps its body perpendicular to the seafloor, ventral side with the funnel pointing forward, and the arms are slightly curved (orally or aborally) to be partly parallel to the seafloor. This is the initial position, from which the feeding behavioural sequence starts (figures 2*a,e* and 3; electronic supplementary material, videos S1–S3). The feeding behavioural sequence is repeated three to seven times in a row (electronic supplementary material, videos S1–S3), and consists of four phases (table 1).

**Table 1.** Feeding behavioural sequence in *Cirroteuthis muelleri*.

phase/character	phase			
	1) spreading	2) enveloping	3) retreating	4) pause^a^
duration, sec, min – max (mean ± s.d.)	4–7 (5.0 ± 0.6)	3–6 (4.4 ± 0.5)	3–5 (4.2 ± 0.4)	1–3 (1.8 ± 0.5)
brachial crown	slowly spreading, enveloping the seafloor	spread to full extent over the seafloor	slowly retracting	retracted, slightly curved
cirri	erect, scanning the seafloor	erect		
distance above the seafloor	equals to cirri length			

^a^Similar to initial position the sequence starts from, see Results.

Duration of the single feeding behavioural sequence is 11–21 s. The feeding observations include a prey capture during the enveloping phase, during the third sequence in PS121-ROV001 (figure 2*j*; electronic supplementary material, video S3) and the seventh sequence in HACON21-ROV019 (figure 2*i*; electronic supplementary material, video S2) in the Fram Strait. Prey capture takes 5–49 s (figure 2*i,j*; electronic supplementary material, videos S2–S6), and involves the octopod becoming more active, pumping all its web sectors and leaning its arms and body towards the seafloor and the prey. This behaviour coincides with rapid rotation of the fins, 24–34 fin stroke cycles min^−1^, presumably to increase the downward pressure on the prey, which is stuck between the octopod and the seafloor. While doing so, the posterior end of the body leans forwards, presumably acting as a counter-balance (figure 2*i,j*; electronic supplementary material, videos S2–S6). In all observations, the octopods take-off from the seafloor after a prey capture (figure 2*k*; electronic supplementary material, videos S2–S6). In one video sequence, we observed mucus with embedded sand and presumably prey remains falling from the octopod's arms after capturing a prey (figure 2*l*; electronic supplementary material, video S4).

#### (ii) Feeding traces

**Figure 4.**
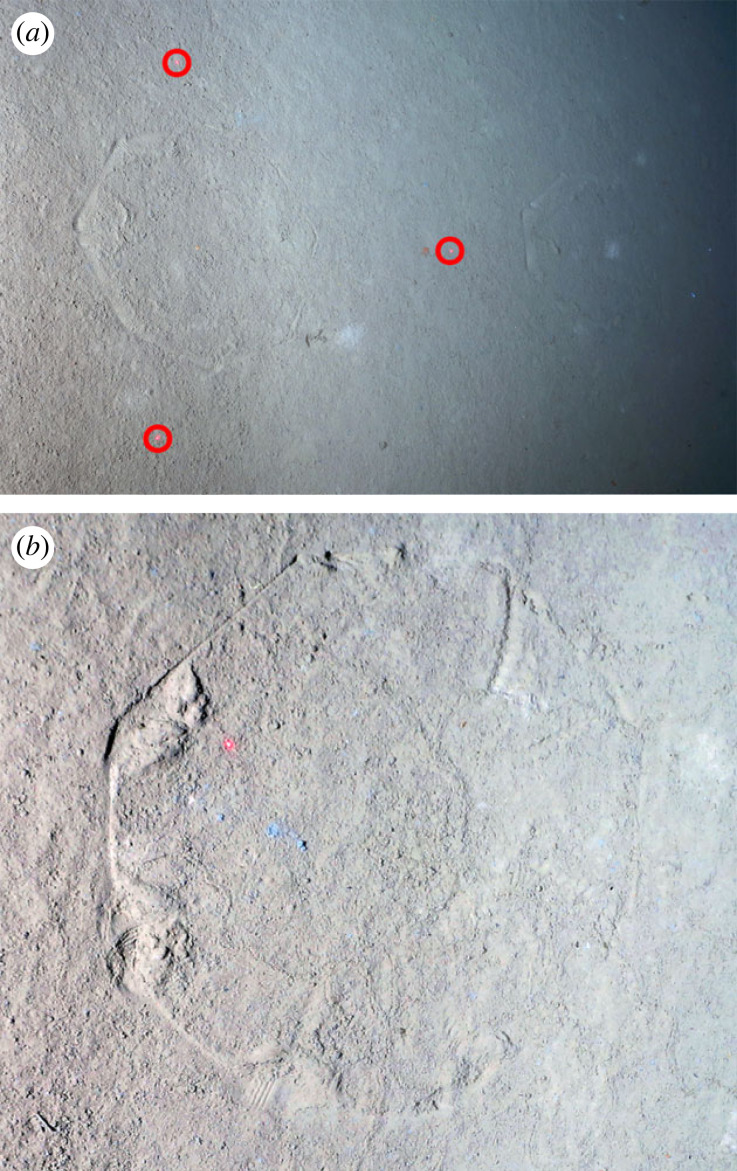
Traces observed on the flanks of the Aurora mound, at depths of 3900–4200 m. (*a*) A pair of octagonal traces, with the one toward the right side of the picture being lower on the Aurora flank slope (as indicated by the light attenuation on that side of the picture. The three red dots (each with red ring placed around for clarity) are spaced 50 cm apart, indicating that the traces are about 30 cm in diameter, matching the sizes of the octopods). (*b*) A close up of another trace. Details from the arms and the curling over of the arm ends slightly on takeoff or prey capture are indicated at the points of web side intersection.

During the OFOBS deployments in the high Arctic, 106 regular octagonal patterns were observed on the seafloor in 92 of the 5100 collected images (figure 4). These patterns often contained indentations from the octopod arms onto the seafloor (figure 4*b*). These traces varied in diameter from 4 to 47 cm, though the great majority were close to the average diameter of 22 cm (s.d. ± 0.09), matching the sizes of the octopods. In one image, the edge of an octopod was just caught in the camera frame (IMAGE: HOTKEY_2019_10_06_at_02_11_10_CP4A6735_3840x5760, online open access data, doi:10.17632/vwtyf2fp5t.1 [45]). Fourteen of the octagonal octopod traces were observed in close proximity to comparably sized additional traces, indicating a localized foraging behaviour before a return to the water column. On several occasions octopods were observed in the lower few metres of the water column by the OFOBS system, either in an umbrella posture or swimming (electronic supplementary material, table S1).

#### (iii) Take-off and fin-swimming (long ROV observations)

The take-off from the seafloor is initiated by a contraction of the brachial crown and a simultaneous single fin stroke cycle (figure 2*k*; electronic supplementary material, videos S2–S6) of 2–4 s, 15–30 fin stroke cycles min^−1^. After the take-off, the octopod performs fin-swimming and rises to 2–3 m above the seafloor, swims towards the ROV and then out of ROV's sight (figure 2*l*; electronic supplementary material, videos S2–S6). The speed of fin-swimming is 7–16 fin stroke cycles min^−1^ (electronic supplementary material, videos S2–S8).

#### (iv) Possible protective reaction

The octopods showed no sign of being scared or disturbed by ROVs, and even approached them very closely on certain occasions (figure 2*m*; electronic supplementary material, video S8). The only possible disturbance, followed by a possible aborted protective reaction, was during PS121-ROV001 (figure 2*n*; electronic supplementary material, video S3). After 24 sec of vertical fin-swimming, the octopod decreased its speed (from 13 to 7 fin stroke cycles min^−1^) and spread its brachial crown moderately for 7 s. However, it resumed the fin-swimming posture and increased speed to the initial velocity thereafter.

#### (v) Take-off, fin-swimming and drifting (PELAGIOS and short ROV observations)

The majority of these observations were recorded from afar. All observations were of short duration, 2–15 s. No signs of survey gear scaring or disturbing the octopods were recorded. There were ten drifting individuals observed, with four additional individuals observed in postures not fitting with drifting (figure 2*o–s*; electronic supplementary material, videos S9–S22). Drifting involves an umbrella posture with the mantle pointing up (30% of observations) or down (70% of observations), where the arms and web are outspread and slightly curved aborally, fins spread (not visible in one observation) and the octopod is not moving (figure 2*o–s*; electronic supplementary material, videos S9–S11, S13, S14, S16, S18–S20, S22). The four non-drifting observations show octopods with their brachial crowns moderately spread (electronic supplementary material, videos S12, S15, S17, S21), fitting pelagic take-off [46]. Drifting is observed at 490–2260 m above the seafloor and fin-swimming is observed at 990–2550 m above the seafloor (electronic supplementary material, table S1).

#### (vi) Quantitative and time comparison between benthic and pelagic observations

Benthic observations occurred at all times of the day except for 08.00–12.00 UTC (electronic supplementary material, table S1). Pelagic observations occurred at all times of the day except for 12.00–16.00 UTC (electronic supplementary material, table S1). A quantitative and statistical comparison of time expenditure in benthic and pelagic environments is not possible due to the different survey methodologies and an imbalanced survey effort.

## 4. Discussion

### (a) Species identification

The observed octopods were identified as *C. muelleri*, the only cirrate so far known from the region [15,28,29]. The absolute and relative body dimensions of the observed octopods (except for fin width in some individuals) fit with the morphometrics of fixed individuals [22] and *in situ* observations [47] of *C. muelleri*.

### (b) Feeding behaviour

This study provides the first *in situ* observations of cirrate octopods feeding behaviour in nature. Several lines of evidence support the conclusion that the observed behaviour of *C. muelleri* on the seafloor involves feeding. First, stomach contents analysis of *C. muelleri* reports a diet of primarily benthic prey [22]. Second, the repeated bottom-associated behaviour of spreading, enveloping and retreating as observed here for *C. muelleri* is different from the near-bottom behaviour of locomotion and pumping reported for *Cirrothauma* cf. *magna* [46]. Thirdly, the release of mucus and potential prey remains and the strong increase in fin-flapping frequency at the end of the behavioural sequence suggests prey handling. Finally, we observed traces as evidence that cirrates interact with the seafloor. In areas of generally low surface perturbation, such as found across much of the Fram Strait [48] and the Aurora mound [49], the characteristic trace shape of the octopod can be left on the seafloor, and remain visible until disturbed or covered. Within this study, the angled flanks of the Aurora mound were marked with many such traces. Additionally, photographic observations of *C. muelleri* in the Central Polar Basin [47] correspond to one of the phases of our suggested feeding sequence. Interestingly, such a seafloor interaction observation was noted in the weekly cruise reports from the PS86 Polarstern expedition to the Aurora mound in 2014, as well as the observation of the trace outlines, but was not investigated further by the onboard team (weekly reports available from PS86: https://www.pangaea.de/expeditions/bybasis/Polarstern). The repeated observations of similar behaviour in different geographical regions, as well as in 2014 and 2019 at the Aurora mound, suggest a common feeding behaviour in *C. muelleri*.

The stomach contents of *C. muelleri* include crustaceans (Calanoida, Mysidacea, Amphipoda, Isopoda and Cumacea) and polychaetes (Polynoidae) [22]. Five of the six taxa are benthic epi- and infauna, and calanoids (and partly amphipods) are hyperbenthic [22,50,51]. These taxa are abundant in the Fram Strait, as both epi- and infauna [52,53]. At the Aurora mound, epifauna was rarely observed in OFOBS images from HACON19, and fauna abundances have not been determined. However, unidentified polychaetes were occasionally observed on the seafloor (see ‘Data accessibility'). Maximum length of crustaceans is 17% of predator's ML (much smaller in case of Calanoida prey), and polychaetes reach 50% ML [22]. The differences in prey size may explain the difference in prey capture duration, where the short capture (5–12 s) may involve crustaceans and the longer captures (49 s) involving large polychaetes. Aquarium observations suggest that the cirrate *Opisthoteuthis* uses cirri to direct small crustacean prey towards its oral cavity using water flows [26]. It is not known how they handle larger prey, such as polychaetes. Compared to *Opisthoteuthis*, the cirri of *Cirroteuthis* are relatively long and with reduced musculature [3,22,23]. The cirri may be used to scan the seafloor for prey during feeding sequences and also help manipulating the entrapped prey towards the oral cavity as in other cirrates [24]. External glands around the lips [22] may facilitate capture or transport of food and explain the release of mucus as observed in this study, and in other cirrates [24].

Metabolic demands and nutritional needs of *C. muelleri* are unknown and hence remain subject of speculation. The metabolic demands of another cold-water species of cirrate octopods, *Stauroteuthis syrtensis* Verrill, 1879 [54], were estimated using aquarium-held individuals and were met by eating 2–30 copepods day^−1^ [55]. Field captured individuals of *S. syrtensis* had from single to ‘many' copepods in their stomachs, as well as occasional mysids and isopods [22,24,55]. It may mean that under natural conditions cirrates consume more prey than when kept alive in the laboratory [55]. Captured individuals of *C. muelleri* from the Arctic had similar numbers of prey as *S. syrtensis* but a more diverse food spectrum [22]. Metabolic rates of *C. muelleri* need to be assessed in an experimental way, using live-caught individuals and published stomach contents data as a baseline [22].

### (c) Pelagic–benthic migrations

*Cirroteuthis muelleri* individuals also spend a portion of their lives in the pelagic realm far off the seafloor. During pelagic video transects, we repeatedly saw *C. muelleri* 500–2600 m above the seafloor. The majority of cirrate observations thus far report individuals close to the seafloor [21,24,46,47,55–59]. However, there are trawl records and observations of Cirroteuthidae 632–3600 m above the seafloor [42,58]. In the pelagic realm, *C. muelleri* drifts motionlessly (umbrella posture), with occasional brief fin-swimming presumably to maintain or change altitude. Drifting cirrates were observed in every one of the six analysed PELAGIOS hauls from two cruises, which were performed at four stations in the Fram Strait (electronic supplementary material, table S1). This coupling with observations from [42,58] suggests that Cirroteuthidae spend a significant portion of their time drifting in the pelagic realm. While we cannot completely exclude that *C. muelleri* opportunistically consumes food in the water column, the body morphology of Cirroteuthidae seems not capable of engulfment of prey in the water column, since their suckers are weak and modified [22,60]. Also individuals move rather slow in comparison to fast moving pelagic crustaceans, or drift motionlessly [46,59]. Moreover, stomach content data only reports benthic prey for *C. muelleri* [22]. This suggests *C. muelleri* feeds primarily on the seafloor, and will have to go all the way down in order to hunt. This extensive pelagic–benthic migration of thousands of metres to the seafloor is unique among cephalopods. Other deep-sea cephalopods either live and forage on the seafloor (benthic incirrate octopods) or live and forage in the pelagic realm (pelagic incirrate octopods, squids and vampire squid) [9,17,61].

Pelagic–benthic migrations are a way to avoid predators and to benefit from benthic productivity and food availability, and are employed also by deep-sea holothurians and fishes [30,31]. There are striking similarities in behaviour and morphology between pelagic–benthic migrants from different phyla. At least six species of deep-sea holothurians and fishes from 11 orders adopt a bentho-pelagic life style as adults [30,31,62]. Their bodies have gelatinous consistencies and relatively large sizes (50–400 mm in length in holothurians and 50–1800 mm in fishes) [30,31,62,63]. Mean body sizes of Cirroteuthidae are between those of bentho-pelagic holothurians and fishes (170–1700 mm TL) [3,22,42–44]. Holothurians and fishes spend the majority of their time in the pelagic realm and feeding on the seafloor is brief (usually < 1 min in holothurians) [30,31,62]. Our observations suggest that for one feeding event, Cirroteuthidae do not spend more than 2.5 min on the seafloor. A benefit of the bentho-pelagic life style is that it allows for reaching feeding grounds that are inaccessible to other deposit-feeders (Holothuroidea), and to escape from physical benthic hazards (turbidity currents, slumping) [30,64]. Passive drifting in deep-sea currents, as found in holothurians and liparid fishes [30,31,65] and here suggested for Cirroteuthidae, may also help to save energy in an environment where resources are patchily distributed. However, the short feeding time on the seafloor of all pelagic–benthic migrating taxa suggests that the threat from predators [30,31] is the main selective pressure for pelagic–benthic migration. Indeed all pelagic–benthic migrants are also prey for a range of predators [3,15,31,66,67]. In particular, predators of cirrates include deep-sea fishes, sharks, and deep-diving toothed whales [3,15]. Depressions left on the abyssal seafloor down to 4260 m depth in the North [68] and South Pacific [69], supposedly by deep-diving toothed whales [68,69], prove these whales threaten deep-sea fishes and cephalopods near the seafloor. Potential predators of *C. muelleri* that are found in high densities in the study area include the Greenland shark and several species of deep-diving toothed whales, e.g. bottlenose and pilot whales [67,70]. Benthic predators are currently known to influence pelagic prey vertical distribution elsewhere [71]. The presence of benthic predators can cause *Pleurobrachia* ctenophores to significantly increase their altitude above the seafloor [71]. Our observations suggest that predation pressure of seafloor-associated predators may be an important but not yet understood driver of vertical distribution in deep-sea fauna.

Several factors underline the importance of the observed pelagic–benthic migrations in energetic coupling between the pelagic and benthic deep sea. *Cirroteuthis muelleri* is one of the most abundant cirrate octopods [3,22]. The densities of these cirrates in the Baffin Bay and likely in our study area are among the highest recorded for cirrates [22]. They prey on abundant and widespread benthic detritophagous, carnivorous and scavenging taxa with relatively small individual sizes [3,22–25,55]. The feeding migration contributes to the entrance of carbon from benthic foodweb to the pelagic foodweb, and this carbon may be channeled further up in the foodweb when cirrates are preyed upon. The actual scale of energy transfer through cirrate octopods in the deep-sea is yet to be assessed.

## 5. Conclusion

Striking morphological and behavioural similarities in bentho-pelagic Cirroteuthidae and several orders of holothurians and fishes suggest that deep-sea bentho-pelagic life styles have appeared independently several times in different phyla. This is an interesting example of a convergent evolution in deep-sea megafauna. The main ecological reasons are likely to avoid the threat from predators and to save energy by using passive transportation with ocean currents. Diel vertical migrations of mesopelagic fauna to the surface layers are the largest animal migrations in terms biomass, number of individuals and contribution to vertical carbon transport [72]. The counter-directed pelagic–benthic migrations by several taxa in the deep sea, which may be relevant for energetic coupling between the pelagic and benthic deep sea, remains to be quantified. These large-scale (over 2.5 km) vertical movements of megafauna should be considered in conservation strategies, and illustrate how deep-sea environments are ecologically connected.

## Data Availability

Videos are available online from Zenodo (https://doi.org/10.5281/zenodo.7728042) [73] and PANGAEA (https://doi.pangaea.de/10.1594/PANGAEA.957196) [74]. The raw seafloor images collected during HACON19 are available from PANGAEA (https://doi.org/10.1594/PANGAEA.943364) [75]. Processed images of seafloor octopod traces, and the one octopod observed on the seafloor during HACON19 with the OFOBS platform, along with tables of trace diameter are available from Mendeley Data (https://doi.org/10.17632/vwtyf2fp5t.1) [45]. All octopod images collected with the OFOBS platform during PS126, MSM95 and MSM108 are available from Mendeley Data (https://doi.org/10.17632/f96cgnjyty.1) [76]. Additional data are provided in the electronic supplementary material [77].
